# Sweating the small stuff: microclimatic exposure and species habitat associations inform climate vulnerability in a grassland songbird community

**DOI:** 10.1098/rsbl.2024.0599

**Published:** 2025-01-22

**Authors:** Jacy S. Bernath-Plaisted, Christine A. Ribic, Benjamin Zuckerberg

**Affiliations:** ^1^ Department of Forest and Wildlife Ecology, University of Wisconsin-Madison,1630 Linden Drive, Madison, WI 53706, USA

**Keywords:** climate change vulnerability, grassland birds, habitat selection, microclimate, microrefugia, nesting success

## Abstract

Assessment of species’ vulnerability to climate change has been limited by mismatch between coarse macroclimate data and the fine scales at which species select habitat. Habitat mediates climate conditions, and fine-scale habitat features may permit species to exploit favourable microclimates, but habitat preferences can also constrain their ability to do so. We leveraged fine-resolution models of near-surface temperature and humidity in grasslands to understand how microclimates affect climatic exposure and demographics in a grassland bird community. We asked: (i) Do species select favourable nest-site microclimates? (ii) Do habitat preferences limit the ability of species to access microclimates? (iii) What are the demographic consequences of microclimatic exposure? We found limited evidence that grassland birds select cooler microclimates, which may buffer eggs and nestlings from extreme heat. Instead, many species appeared constrained by vegetation preferences. While facultative generalists displayed flexibility to use denser vegetation that provided buffering from high temperatures (>39°C), most obligate species nested in more exposed microclimates. Nesting success in facultative species was not well explained by microclimate, but success in specialized grassland obligates declined with increasing microclimate temperatures. These findings illustrate how microclimate and habitat use can interact to influence the potential vulnerability of species to future climate change.

## 1. Introduction

Species are increasingly subjected to unprecedented extremes of temperature and precipitation [1,2]. Quantifying both current and future exposure of species to climate has therefore become critical to assessing the vulnerability of species to future change [3]. Until recently, ecologists have relied on coarse resolution (typically ≥1 km^2^) macroclimate data to model species–climate relationships [4,5]. However, these data often do not reflect the climate conditions that species experience in the environment or the scales at which they may select habitat [5–7]. This is because variations in vegetation, topography and biophysical processes near the Earth’s surface create heterogeneity in climate at much finer scales [8,9].

Habitat is an important mediator of climate, and species habitat associations may influence their exposure to climate [10,11]. For example, the dense canopies of old-growth forests have a high capacity to moderate heat and ameliorate species declines in response to high temperatures [12,13]. By contrast, species in open systems may be more vulnerable to high temperatures due to the absence of such forest canopy [14,15]. Yet, even within open systems, microclimate heterogeneity may allow species to adaptively select habitats that confer thermal benefits and enable them to persist under stressful climate conditions [16,17]. Over longer time scales, such microrefugia can prevent species extirpations with shifting climate regimes [18]. However, despite the potential availability of microrefugia, species may be constrained by habitat preference [19] or competing selective pressures [20] that prevent them from accessing refugia. Our understanding of species vulnerability to climate change could be improved not only by better integration of microclimate data but also a broader understanding of how species select microclimates and the consequences of such selection. Unfortunately, detailed, empirically collected microclimate data remain rare [21], though interest in the topic is growing [22–24]. Although software and tools capable of mechanistically modelling microclimate are becoming increasingly powerful [25,26], these models are still limited by the availability of high-resolution remote sensing data and gridded climate products needed to inform microclimates [27]. Finally, few studies have examined the full linkages between microclimates, habitat selection and demographic consequences [12,14,28,29].

**Figure 1.**
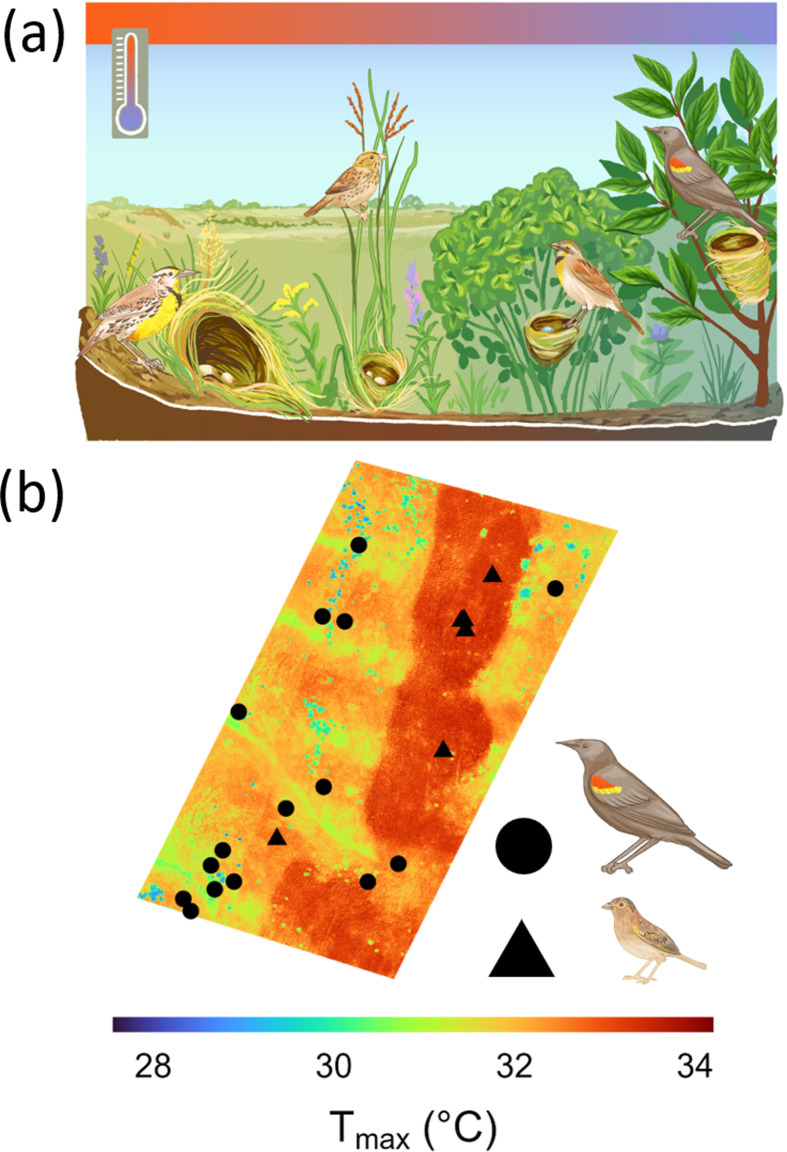
The hypothesized influence of species habitat associations on microclimate temperature exposure at the nest (a). Red-winged Blackbird nests (circles) and Grasshopper Sparrow nests (triangles) overlaid with microclimate predictions of daily maximum temperature (b).

We sought to advance the ecological understanding of microclimates by leveraging fine-resolution spatiotemporal models of near-surface temperature and humidity in North American temperate grasslands [30], and nesting data for a community of grassland songbirds. Grassland birds are among the most steeply declining avian assemblages in North America [31], and their demographics are sensitive to climate and weather [32,33]. Grassland birds also display strong interspecific variation in evolved vegetation preferences [34,35]. In particular, grassland-obligate species that depend exclusively on grassland habitat during the breeding season and facultative species, which are more generalist and, though found in grasslands, can use other open breeding habitats such as wetland, shrubland and savanna [34,36], may be exposed to different microclimates as a consequence of contrasting vegetation preferences. Facultative species often display greater flexibility to select nest sites in dense vegetation such as shrubs and forb clumps [37] and often respond differently to habitat management (e.g. vegetation structure and mechanical treatments) than obligate species [38]. Consequently, despite co-occurring within the same grassland ecosystems, grassland bird species may differ in microclimate exposure because of variations in vegetation height and density at the nest site, which may affect shade and evaporative cooling [23,24,30] (figure 1*a*).

Selection of the nest site represents a habitat choice with strong consequences for reproductive success in bird species [39], and in open grasslands, nest-site microclimate plays an important role in buffering eggs and nestlings from lethal climate conditions, such as exposure to extreme heat and aridity [23,24,32]. Elevated temperatures can negatively affect nesting success directly through mechanisms such as evaporative water loss (EWL) and hyperthermia, which may cause nestling mortality [40] and egg unviability [41]. Temperature and humidity may also interact to affect the risk of nestling mortality from climate exposure. For example, high temperatures and low humidities may increase rates of EWL, but high temperatures and saturating humidities can impair the dissipation of heat [42]. Microclimates may also affect success indirectly through behavioural trade-offs. For example, nest sites that are exposed to temperatures above critical thresholds may require adults to spend excessive time thermoregulating the nest at the expense of nestling provisioning [43].

Here, we addressed three questions exploring the importance of microclimates to breeding birds. (i) Does habitat heterogeneity allow grassland birds to select nest sites with beneficial microclimates? We hypothesized that, given the sensitivity of grassland birds to elevated temperature and drought, species should select nest sites that reduce exposure to hot and dry conditions. (ii) How do species’ habitat preferences affect their ability to select optimal microclimates? If facultative species display greater flexibility to use dense vegetation, we hypothesized that they would receive more buffering from hot and dry conditions than obligate species nesting in sparser cover [14]. (iii) What are the consequences of microclimatic exposure for species? We hypothesized that if exposure to hot and dry conditions increases the probability of eggs and nestlings experiencing hyperthermia and EWL, or require adults to spend greater time and energy thermoregulating the nest, success should be lower for nests with greater exposure to these conditions. We also predicted that obligate species would show greater demographic sensitivity to temperature than facultative species due to nesting in more exposed habitat.

## 2. Methods

### (a) Study system

We collected data between 10 May and 10 August 2020−2022 at four planted grasslands in southern Wisconsin, USA (~42° 55′ 11.45′′, –89° 50′ 5.91′′). Two of the four sites were dominated by a cool-season grass species, smooth brome (*Bromus inermis*), while the remaining sites were tallgrass prairie restorations planted with a diversity of native grasses and forbs. The most abundant grassland songbirds nesting at our sites included five obligate species: Bobolink (*Dolichonyx oryzivorus*), Dickcissel (*Spiza americana*), Eastern Meadowlark (*Sturnella magna*), Grasshopper Sparrow (*Ammodramus savannarum*) and Henslow’s Sparrow (*Centronyx henslowii*), as well as three facultative species: Common Yellowthroat (*Geothlypis trichas*), Field Sparrow (*Spizella pusilla*) and Red-winged Blackbird (*Agelaius phoeniceus*). All of these species are migratory and returned to our sites in April and May. Nest initiations (e.g. date of first egg laid) by species for all nests monitored in our study are available in electronic supplementary material, figure S1, and procedures used to estimate initiation date are in the electronic supplementary material. Detailed descriptions of species nesting associations, as well as additional details on study sites and weather conditions, are also available in the electronic supplementary material.

### (b) Microclimate data

The microclimate estimates used in our analyses are generated from recently developed spatiotemporal random forests models of near-surface grassland microclimate [22]. These models were produced using hourly temperature and humidity observations from iButtons deployed systematically across study sites. These data were paired with UAS-collected (unpiloted aircraft systems, e.g. drones) LiDAR (light detection and ranging) and multi-spectral imagery of sites that were used to create spatial predictors of microclimate. These included vegetation height, NDVI (normalized difference vegetation index), distance to wooded edge, elevation, topographic positioning, slope, aspect and hill shade. Local weather data were also included as predictors in the models so that, for any day of the season and at any location within our study sites, an estimate of near-surface microclimate could be generated at a 60 cm resolution. We used the model to produce estimates of daily maximum (T_MAX_), minimum (T_MIN_) and average (T_AVG_) air temperatures as well as daily maximum (VP_MAX_), minimum (VP_MIN_) and average (VP_AVG_) vapour pressures. However, we chose to focus primarily on T_MAX_ and VP_MIN_ as we felt extremes of heat and dry best represented conditions that would affect nesting grassland bird demographics [32,33,44]. For example, 39°C is often identified as a lethal temperature threshold for birds, and nesting success may begin declining above 35°C [32,40]. The two models of T_MAX_ and VP_MIN_ had cross-validated accuracies of < 2°C and < 0.10 kPa, respectively. Additional modelling details are in the electronic supplementary material. Given the challenges and uncertainty involved in collecting remotely sensed grassland data and the lack of detailed information on grassland microclimates in the literature, we felt it was most conservative to view these models as representing relative differences in exposure to extreme conditions between locations at a fine scale, and thus we did not attempt to precisely model operative temperatures as mechanistic microclimate models often do [25].

### (c) Nesting data

We searched for nests daily between 15 May and 15 July both systematically (rope dragging and walking) and behaviourally [45,46]. We marked nests non-invasively with survey flagging placed 5 m to the north and south of the nest. We monitored nests at 2- to 5-day intervals (typically 3) and recorded nest contents and activity at each visit. Nests were defined as successful if they fledged at least 1 young and failed if they did not (details in electronic supplementary material). We also calculated the initiation date for each nest; in cases where initiation date was not known from finding the nest during construction or back-dated from hatch date, dates were estimated using clutch size, incubation lengths and known activity periods (details in electronic supplementary material). We made grassland vegetation measurements at each nest within 1 week of termination and measured visual obstruction reading (VOR), a common grassland measurement that describes vegetation height and density [47,48], in four cardinal directions using a Robel pole. We used a sub-metre accuracy GPS unit (Trimble Geospatial, CA, USA) to measure each nest location within < 5 cm.

### (d) Data analysis

All analyses were performed in Program R [49]. To assess the effect of microclimate on nest-site selection in grassland birds, we used conditional logistic regression (details in electronic supplementary material) implemented in the *survival* package [50]. We sampled random points within 50 m of matched nests to represent locations that were realistically accessible to each nesting pair. We estimated microclimate using the spatial models described above for 7 days before the estimated initiation date [24], defined as the laying of the first egg, and averaged these estimates to represent microclimate conditions during the week in which selection likely occurred. For each species, we constructed univariate conditional logistic regression models comparing microclimate at nest sites with five nearby random points (< 50 m; electronic supplementary material).

To explore how differences in species habitat associations influence their subsequent exposure to microclimate, we compared mean nest-site VOR among species using a single-factor ANOVA for unequal variances with a Games–Howell test corrected for multiple comparisons. To determine if vegetation conditions at the nest site moderate exposure to high temperatures and dry conditions, we used the *gamlss* package [51] to perform a zero-inflated beta regression (details in electronic supplementary material) relating nest-site VOR to the proportion of active days for which each nest experienced T_MAX_ > 39°C and VP_MIN_ < 0.915 kPa. We used these calculations as metrics of heat and dry exposure for each nest. We chose our temperature threshold because air temperatures around and beyond 39°C can be increasingly lethal for eggs, nestlings, and even adult birds [40,52]. For vapour pressure, we could find no similar justification for a threshold in the literature, so we used the 20th percentile of estimated vapour pressures to characterize the driest conditions that nests were exposed to during our study. We included initiation date (e.g. indicating when the nest was active) and year in these models to account for broader variability in climate. Finally, to compare exposure to microclimate conditions between nests of obligate and facultative species, we also performed a Wilcoxon sum-rank test comparing heat exposure and dry exposure (as we define them above) between the two groups.

We used logistic exposure [53] to examine the effects of microclimate on nesting success. We first conducted a preliminary analysis to determine if body mass interacted with air temperature to affect nesting success. Body size plays an important role in thermal regulation [40] and is often tested for in microclimate studies [24]. Our focal species ranged in average body masses from 10 g (Common Yellowthroat) to 92 g (Eastern Meadowlark). However, we found no significant interaction between T_MAX_ and body mass (*p* = 0.314), so we did not include body mass in subsequent analyses. We analysed obligate and facultative species in separate models to determine if they responded differently to microclimate conditions, and we tested both time-specific and cumulative effects of microclimate, as well as the interaction between temperature and humidity, which can influence hyperthermia and EWL rates [42]. For each species group, we parametrized seven models:

1. T_MAX_ + Date + Year (Daily temperature model)
2. VP_MIN_ + Date + Year (Daily vapour pressure model)
3. T_MAX_ X VP_MIN_ + Date + Year (Daily interactive model)
4. EXP_T+Date + Year (Cumulative temperature exposure model)
5. EXP_VP+Year (Cumulative dry exposure model)
6. EXP_T X EXP_VP + Year (Cumulative interactive exposure model)
7. Visit date + Year (null model)

We were unable to include dry exposure (EXP_VP) and visit date (e.g. the date the nest was checked by an observer) in the same models because they were correlated (all variables were checked for correlation). We compared models using AIC*_c_* model selection and selected the model with the lowest AIC*_c_* score and considered models to be competitive for ΔAIC*_c_* < 2.00 [54]. We considered parameters to be informative *only* if 90% confidence intervals did not overlap zero [55] *and* the model containing the parameter of interest occurred in the top model set [54]. All data and code supporting the results presented herein are available from the Dryad Digital Repository [56].

## 3. Results

**Figure 2.**
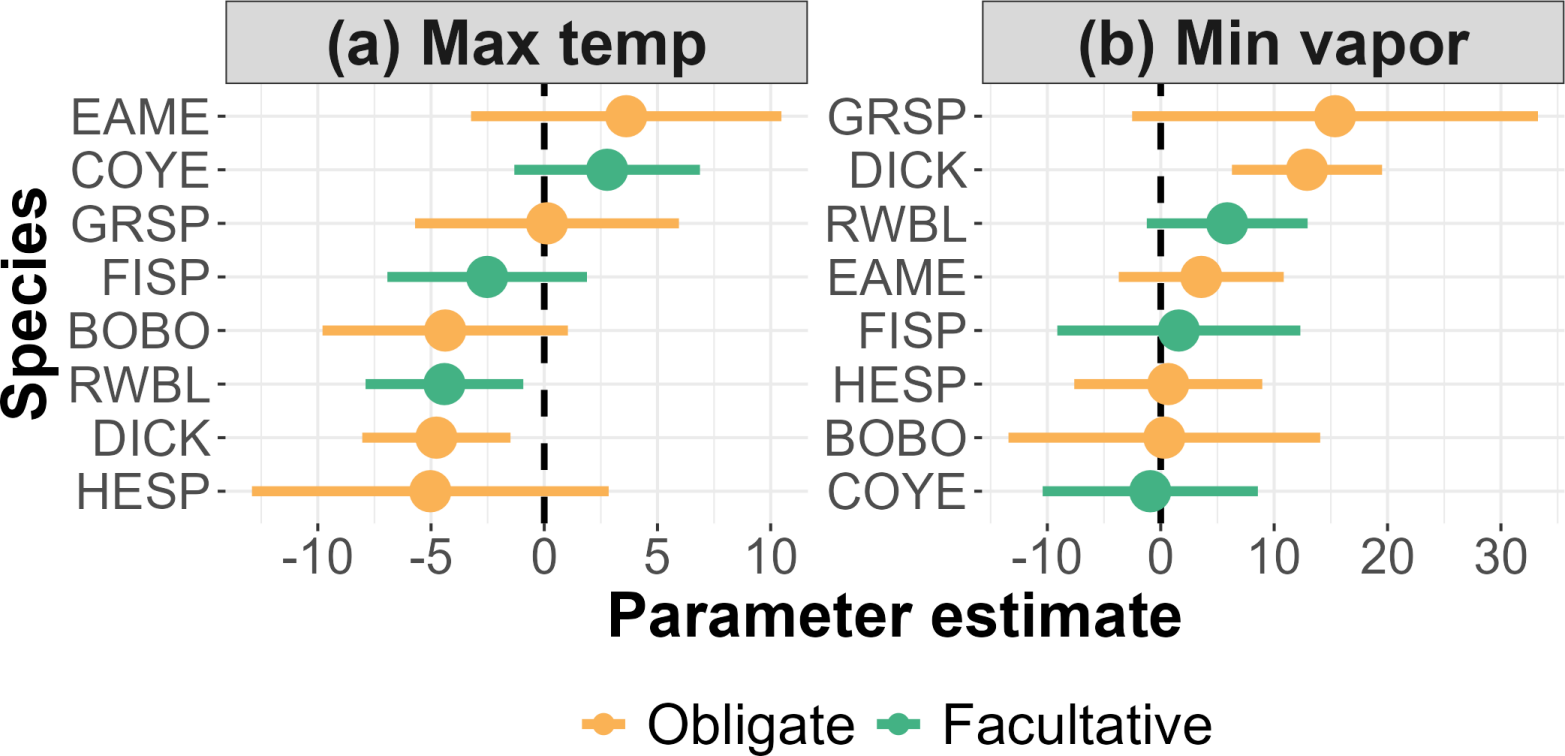
Dickcissel (DICK) and Red-winged Blackbird (RWBL) showed evidence of selection for cooler microclimates relative to random locations (a), and Dickcissel also avoided dryer conditions (b). Error bars denote 95% confidence intervals. Species codes and sample sizes: Red-winged Blackbird (RWBL) = 47, Eastern Meadowlark (EAME) = 37, Dickcissel (DICK) = 33, Common Yellowthroat (COYE) = 21, Bobolink (BOBO) = 17, Henslow’s Sparrow (HESP) = 17, Field Sparrow (FISP) = 12, Grasshopper Sparrow (GRSP) = 11.

Comparison of microclimates at nest sites with nearby random locations revealed that two shrub-associated species, grassland obligate Dickcissel (*n* = 33) and facultative species Red-winged Blackbird (*n* = 47), selected nest sites that were characterized by significantly cooler temperatures (lower T_MAX_) relative to available sites (*p* = 0.004, *p* = 0.013, respectively; figure 2*a*); Dickcissel also selected significantly more humid sites (higher VP_MIN_) relative to random locations (*p* < 0.001; figure 2*b*). Six other species showed no statistical evidence of nest-site selection with respect to T_MAX_ and VP_MIN_ (electronic supplementary material, table S1). Additional results for all microclimate variables that we modelled can be found in electronic supplementary material, figure S2 and table S1.

**Figure 3.**
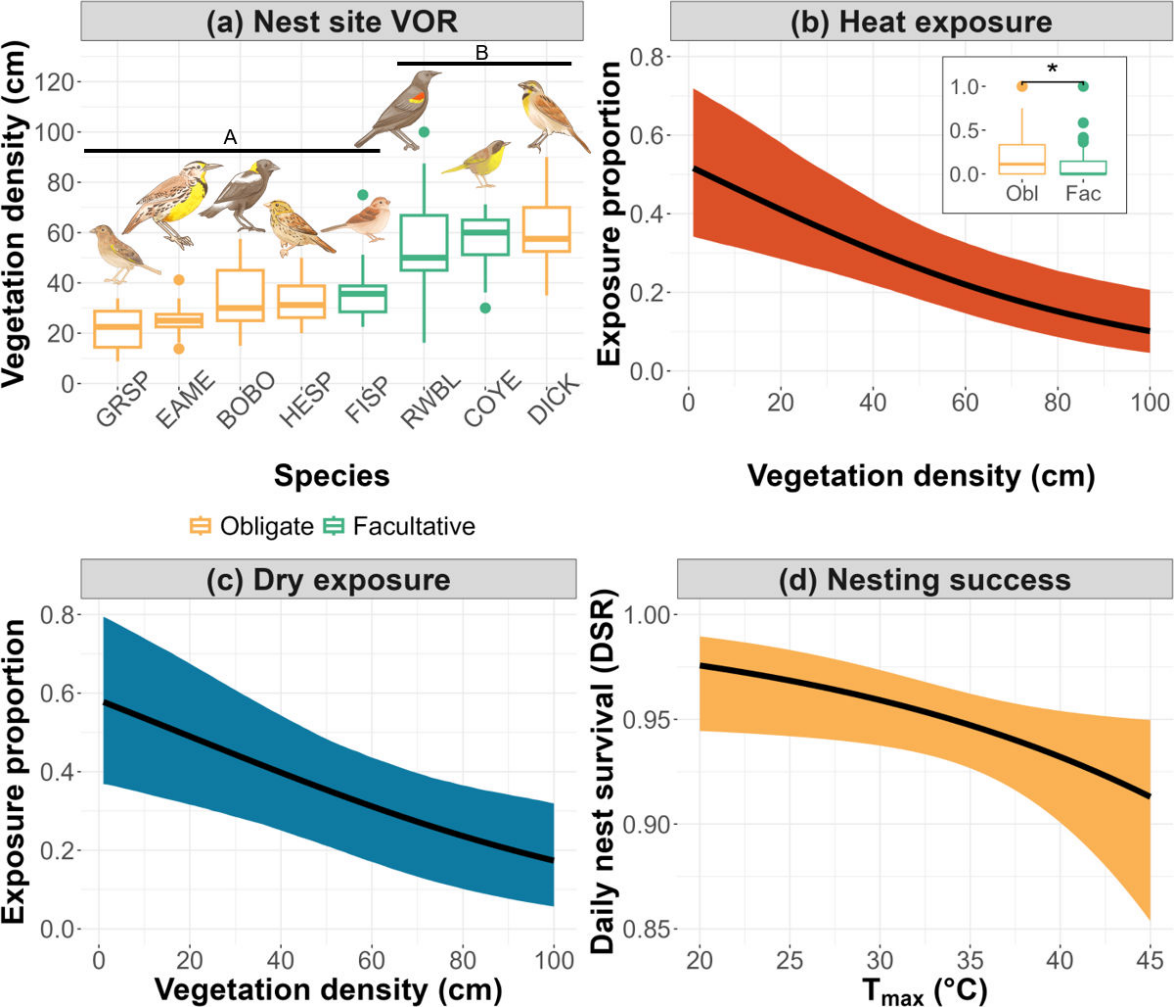
Facultative and shrub/forb nesting species nested in locations with significantly (*p* < 0.050) higher vegetation density (VOR) than ground-nesting obligate species (a). High vegetation densities subsequently reduced the proportion of days that nests were exposed to high temperatures and facultative nest sites were significantly (p = 0.002) less exposed to heat than those of obligates (b). Similarly, vegetation density also reduced exposure to drier conditions (c). Daily nest survival (DSR) of grassland obligates declined with elevated daily maximum temperatures (d). Shading denotes 95% confidence intervals around predictions.

Though most species did not display evidence of microclimate selection, species experienced differing microclimatic exposures as a consequence of habitat association (figure 1*b*). Ground-nesting obligates like Eastern Meadowlark (*n* = 37) and Grasshopper Sparrow (*n* = 11) placed their nests in sparse vegetation compared to facultative species like Common Yellowthroat (*n* = 21) and Red-winged Blackbird (*n* = 47), as well as the shrub-nesting grassland obligate Dickcissel (*n* = 33), all of which placed their nests in areas with significantly greater vegetation density (*F* = 35.71, d.f. = 7, *p* < 0.001; figure 3*a*). Obligate and facultative nest sites had mean VORs of 37.34 cm (s.d. = 18.02) and 52.53 cm (s.d. = 16.57), respectively. Additional nest-site vegetation data can be viewed in electronic supplementary material, figure S3.

Differences in nest-site vegetation had microclimatic consequences for grassland birds. For nests of all species combined (*n* = 195), nest sites within higher vegetation density experienced significantly fewer days of exposure to extreme air temperatures (*β* = −0.44, s.e. = 0.10, *p* < 0.001, *R*^2^ = 0.47; figure 3*b*) and dry conditions—minimum vapour pressures below the 20th percentile for our study (*β* = −0.38, s.e. = 0.11, *p* = 0.001, *R*^2^ = 0.44; figure 3*c*). For example, over a 22 day nesting cycle, the average dickcissel nest with a VOR (e.g. density) of 60 cm would experience ~4 fewer days of exposure to approximately 39°C or greater than the average Grasshopper Sparrow nest with a VOR of 20 cm. We also found that, on average, nests of grassland obligate species (*n* = 115) experienced a significantly (*W* = 5738, *p* = 0.002) higher proportion of heat exposure days than facultative species (*n* = 80). However, there was no difference in proportion of dry exposure days between these groups (*W* = 4939, *p* = 0.377). Full model results for beta regression of heat and dry exposure days against nest vegetation density can be found in electronic supplementary material, tables S2 and S3.

We found that microclimate exposure at nest sites had reproductive consequences for breeding grassland birds. For grassland obligate species (*n* = 115), T_MAX_ was the top model explaining daily nest survival (ΔAIC*_c_* = 2.32, *W_i_* = 0.51; electronic supplementary material, table S4), and higher temperatures negatively affected success (*β* = −0.29, LCI = −0.51, UCI = −0.06; figure 3*d*; electronic supplementary material, figure S6). However, for facultative species (*n* = 80), VP_MIN_, T_MAX_, and the null model were all competitive (electronic supplementary material, table S4), but the only predictor in these models with confidence intervals not overlapping zero was date (electronic supplementary material, table S5). Thus, we observed no similar effect of T_MAX_ on nesting success in facultative species.

## 4. Discussion

Grassland birds are considered highly vulnerable to climate change [57], due to demographic sensitivity to climate [32,33,58], and association with grassland habitats that are exposed to extremes of heat and drought [2,59]. Near-surface temperatures in our study and those measured in other grassland environments often reach temperatures well in excess of 35°C [30,60,61], and though there is relatively little research available on grassland microclimates, it is likely that grasslands in warmer locations around the globe experience even greater extremes. Under climate change, 40% of all land vertebrates are projected to experience thermal extremes beyond anything in their evolutionary histories by the end of the century [2]. Thus, habitat refugia may become increasingly important to species [62].

Although grasslands lack the temperature buffering capacity of forests [14,15,60], fine-scale variation in vegetation density and microtopography in grasslands can still create significant thermal diversity [30]. Theoretically, breeding grassland birds could moderate exposure to hostile climate conditions through the selection of thermally buffered nest sites [23,24]. However, we found that grassland birds in our study appeared largely unable to access the coolest microclimates available. Out of eight focal species, only Dickcissel and the facultative species, Red-winged Blackbird, selected nest sites that were significantly cooler than random locations. Both species are often shrub-associated with known preferences for dense vegetation [37,63,64], and it appears likely that—even for these species—cooler nest sites were a consequence of habitat association rather than selection for microclimates *per se*.

Consistent with previous work, nesting success for grassland obligates declined with high ambient temperatures [32]. Given this demographic sensitivity to temperature, adaptive selection theory predicts that grassland birds should have selected thermally buffered nest sites that were ostensibly available to them [65]. However, there are many ecological and methodological reasons that researchers often fail to detect such adaptive selection in nesting birds [39]. These range from explanations as simple as small sample sizes to the complexities of lifetime reproductive success and carry-over effects to other life-history phases, such as post-fledging survival [39]. A limitation of our study is that we were not able to explicitly test such alternative hypotheses, but evolved habitat preferences and competing selective pressures provide a likely explanation for constraints on microclimate selection in nesting grassland birds.

Predation is typically the largest source of nest mortality in breeding birds, particularly in grassland and shrubland habitats where ~40% of nests are predated [66–68], and thus predation risk is a strong selective force shaping avian life histories [66]. Grassland bird communities are characterized by distinct preferences for microhabitat structure, and individual species are often restricted to a narrow range of vegetation densities [34,35,69]. These preferences likely evolved under selective pressure both to reduce interspecific competition for nest sites and to escape predation risk by reducing nest density and diversifying the search image of nest locations [69,70]. Therefore, grassland birds may be constrained in microclimate selection either by perceived predation risk (e.g. nesting in sparse habitat may increase predator detection, but also exposure to heat and dry) [20,29], or by habitat preferences shaped by selective pressures throughout their evolutionary histories (e.g. niche segregation and density effects) [70]. Regardless of mechanism, grassland obligate birds with strong preferences for relatively sparse vegetation cover appear to lack the behavioural flexibility to access cooler microclimates found in denser vegetation within the same grassland areas. There were clear microclimatic consequences associated with these habitat preferences, and high vegetation density at nest sites consistently reduced exposure to hot and dry conditions. As a result, nests of facultative species were exposed to significantly fewer days exceeding potentially lethal air temperatures than those of obligate species. This finding has implications for understanding interspecific differences in vulnerability to climate change and suggests that ground-nesting grassland obligates may be more threatened by future climate extremes than shrub-associated and facultative species.

Microclimates and microrefugia play an important ecological role in mediating the effects of climate change on animal species [12,13,18,21], and understanding how they influence species habitat use and demographics can better inform our understanding of vulnerability [71]. Our results are significant not only because they demonstrate how microclimates can influence exposure and demographics even in grasslands, but also because they highlight how species responses to microclimate may be individualistic and contingent on habitat preferences. As the ecological relevance of microclimates has increasingly been recognized, maintaining thermal heterogeneity across landscapes has also emerged as a climate adaptation strategy [10,17,72]. While this is an important goal and one that may help to support biodiversity under climate change, it is also important to realize that not all species will benefit equally. Specialist species with inflexible habitat preferences may be constrained in their ability to benefit from thermal diversity, and such species will require alternative management strategies for conservation in a changing climate.

## Data Availability

The data and code supporting the results presented herein are available from the Dryad Digital Repository [56]. Supplementary material is available online [73].
